# Enhanced Low-Velocity Impact Properties for Resin Film Infusion-Manufactured Composites by Flow-Control Approach

**DOI:** 10.3390/polym13193431

**Published:** 2021-10-06

**Authors:** Juan-Antonio Almazán-Lázaro, Elías López-Alba, Sebastian Schmeer, Francisco-Alberto Díaz-Garrido

**Affiliations:** 1 Department of Mechanical and Mining Engineering, Campus Las Lagunillas, University of Jaen, 23071 Jaén, Spain; elalba@ujaen.es (E.L.-A.); fdiaz@ujaen.es (F.-A.D.-G.); 2 Leibniz-Institute for Composite Materials (IVW), 67663 Kaiserslautern, Germany; sebastian.schmeer@ivw.uni-kl.de

**Keywords:** composite, reinforced, polymer, manufacturing, impact, optimization, stitching

## Abstract

The optimization of the mechanical properties of composite materials has been a challenge since these materials were first used, especially in aeronautics. Reduced energy consumption, safety and reliability are mandatory to achieve a sustainable use of composite materials. The mechanical properties of composites are closely related to the amount of defects in the materials. Voids are known as one of the most important defect sources in resin film infusion (RFI)-manufactured composites. Minimizing the defect content leads to maximized mechanical properties and lightweight design. In this paper, a novel methodology based on computer vision is applied to control the impregnation velocity, reduce the void content and enhance the impact properties. Optimized drop-impact properties were found once the impregnation velocity was analyzed and optimized. Its application in both conventional and stitching-reinforced composites concludes with an improvement in the damage threshold load, peak force and damaged area. Although stitching tends to generate additional voids and reduces in-plane properties, the reduction in the damaged area means a positive balance in the mechanical properties. At the same time, the novel methodology provides the RFI process with a noticeable level of automation and control. Consequently, the industrial interest and the range of applications of this process are enhanced.

## 1. Introduction

Composite materials are widely used in industries such as transportation and energy, where high performance in mechanical properties is often required. Depending on the production rate and part complexity, different types of processes are available to manufacture composite materials. Liquid composite manufacturing (LCM) techniques are characterized by using the infusion or injection of the resin into a mold once the initial preformed dried fibers are placed inside. The performance of the main processes in this group—including the resin transfer molding (RTM), RTM Light and resin film infusion (RFI)—is strongly dependent on how the dry fibers are impregnated. In all of these, repeatability, safety and cycle times must be optimized to meet the needs of an increasingly demanding industry and the development of the competitiveness of the means of production [1]. Increasing the level of component integration and process automation is another of the current objectives of the composite materials’ industry, so as to reduce the manufacturing, handling and assembly times [2].

RFI is a cost-effective and high-performance process of manufacturing long-fiber-reinforced composites. It is often used to manufacture large-size [2] and highly complex components [2,3] in structural construction [4] for repairing [5] or joining [6]. However, it is not widely used for high-volume production because the process is labor-intensive. Currently, this process shows low automation levels in addition to most of its variables not being properly controlled or being randomly selected. In addition, the potential to optimize the mechanical properties have been preliminary reported [7,8]. These studies highlight the influence of the impregnation velocity on the void content which affects the mechanical properties. Therefore, stitching, as a reinforcement technique, can be used to increase the process and properties’ performance, even though it reduces the in-plane properties. This helps keep the preforms during the process in addition to the fact that it also has influence on the processability and mechanical properties—especially the out-of-plane properties [9]. These additional reinforcements modify the impregnation features, and it should be considered for the process controlling.

The RFI process is based on a one-side mold in which long dry fibers are placed before adding a side-sealed bag. Then, the vacuum is applied in an inlet while the catalyzed resin pot is plugged into the hermetic bag. The pressure difference between the atmosphere and the applied vacuum promotes a flow through the initially dry fibers. The process could be modeled by the Darcy equation for porous media (Equation (1)):

(1) $$\vec{q}=-\nabla P\cdot \frac{\overset{=}{K}}{\mu }$$

where $\vec{q}$ is the volume average flowrate, μ is the dynamic viscosity, *P* is the local pressure and $\overset{=}{K}$ is the permeability tensor. In the unidimensional case, the impregnation velocity can be assessed at each position by Equation (2) where Δ*P*/Δ*x* is the applied pressure gradient, *K* is the unidirectional permeability and Ø is the media porosity:

(2) $$u=\frac{∆P\cdot K}{∆x\cdot \mu \cdot \emptyset }$$

The impregnation velocity, which is shown in Equation (2), is a key process parameter to be considered in optimizing a composite’s mechanical properties. This velocity is often randomly set in the industry and due to the physics under the process, it continuously changes as the impregnation goes further.

One of the most common source of voids is the unbalanced flow in dual-scale preforms [10,11]. Two flows occur during the impregnation of dual-scale preforms: the macroscopic flow is managed by the external applied pressure and flow across the gaps among yarns; and the microscopic flow is managed by capillary effects because of the small gaps between fibers. Both microscopic and macroscopic flows simultaneously take place during the impregnation process [12]. Commonly, one of these flows is predominant over the other. As a result, one of these flows will be faster than the other, and macrovoids or microvoids are likely to be generated. Additionally, as the velocity continuously changes as the flow goes further, the mechanical properties are not spatially homogeneous. In this context, even the most demanding sectors must accept a certain amount of voids: between 1% and 2% in the aeronautical sector [13]; and approximately 5% for other applications [14]. 

On this subject, some preliminary works have demonstrated the influence of impregnation velocity on tensile properties, such Leclerc et al. [15] for RTM and Almazán et al. [7] for RFI. Ideally, the impregnation velocity should be kept at constant and optimum values during the whole process to minimize the void content and maximize the mechanical behavior. Nevertheless, there are no available data on the effects on impact properties, for neither RTM nor RFI. Then, these values can be controlled to optimize some specific property of the materials.

Composite materials may be subjected to impact loads in many applications. Although these materials show high in-plane properties, they are sensitive to out-of-plane loads. Indeed, impact loads usually lead to delamination, which is the main damage mode in composites [16]. Numerous recent studies have highlighted the source and the importance of the impacts of loads on pipes [17], aircrafts [18,19] and vessels [20], mainly during its use or maintenance. Multiple impacts have also studied in some works to analyze the damage accumulation after repeated impacts [21]. In this context, it is very important to consider the impact behavior of composite materials during the material design. Then, a low-velocity impact test was used as a reference to optimize the mechanical behavior of the studied composites. Parameters such as the maximum impact force (*F_m_*), the critical force (*F_c_*) and the damaged area have been considered in some studies [22,23] as indicators to improve the low-velocity impact behavior. For low-velocity impact loadings, the work of Shyr et al. [24] summarized the different damage mechanisms in composite materials. The induced damage is a combination of three mainly failure modes: delamination, matrix cracking and fiber breakage. Fiber breakage mainly occurs at the impact face by compression and buckling, and at the back face as a consequence of tensile stress. Delamination and matrix cracking take place in the matrix, as reported by Robinson et al. [25] and Kumari et al. [26]. This means that the impact properties are strongly dominated by the matrix and influenced by its mechanical properties and possible defects. The matrix plays a highly relevant role in damage generation, propagation and energy dissipation. Since voids are located in the matrix, the study of the matrix and its defects is highly relevant.

With the aim of exploring the effects of flow front velocity in a variety of materials, both stitched and unstitched materials have been used in this work. As a reinforcement, stitching is known as a cost-effective method of improving the out-of-plane mechanical properties of composites. For impact events, the damaged area was reduced by up to 40% when stitching was added [27,28]. Stitching reduces the interlaminar crack propagation as it works as a bridge between the plies, increasing their strength. As reported by some authors such as Rieber et al. [29] and Rimmel et al. [9], stitching also has a strong influence on the permeability and processability of laminates. Then, both stitched and unstitched laminates were taken into account since stitching can modify the void content and consequently the mechanical properties.

In this work, low-velocity impact tests were performed, occurring at velocities below 10 m/s [30]. Subcritical, critical and supercritical impacts can be observed depending on the applied energy and material response. The critical level corresponds to the damage threshold, the energy at which internal fracture damage starts [31].

The goals of this study were focused on finding the optimal impact properties by optimizing the impregnation velocity during the infusion. In all cases, the impregnation velocity should be kept constant during the whole impregnation process to avoid spatial variation in properties. Once the optimum velocity is known, the optimum parameters are compared to those in laminates which include a transversally stitched reinforcement. To perform the optimization, some key indicators are considered, such as the maximum impact force and damaged area. The flow front velocity and its relationship with the material performance were also analyzed and controlled afterwards. An application based on a computer-vision system was developed as a tool to measure and automatically control the velocity value. This encourages the RFI process level of automation and control in order to reduce the variability from external factors and maximize the results.

## 2. Materials and Methods

### 2.1. Flow Front Controller

The flow controller is a key part of this implementation to keep the flow front velocity at constant values and maximize the mechanical properties. From the unidimensional Darcy model (Equation (2)), when an additional pressure loss (e.g., controlled valve) is added, the velocity is consequently modified. Then, the Darcy equation (Equation (2)) can be rewritten as shown in Equation (3), where $∆P_{valve}$ is the added pressure loss:

(3) $$u=\frac{∆P_{pump}-∆P_{valve}}{∆x}\cdot \frac{K}{\mu \cdot \emptyset }$$

Once the optimum velocity is fixed as a target, $u=u_{opt}$, the evolution of $∆P_{valve}$ can be assessed to maintain this optimum flow front velocity. Then, $∆P_{valve}$ should follow the expression shown in Equation (4). Similarly, the time dependency can also be evaluated as shown in Equation (5):

(4) $$∆P_{valve}=∆P_{pump}-\frac{\mu \cdot \emptyset }{K}\cdot u_{opt}\cdot ∆x$$

(5) $$∆P_{valve}=∆P_{pump}-\frac{\mu \cdot \emptyset }{K}{\left(u_{opt}\right)}^{2}\cdot t$$

**Figure 1:**
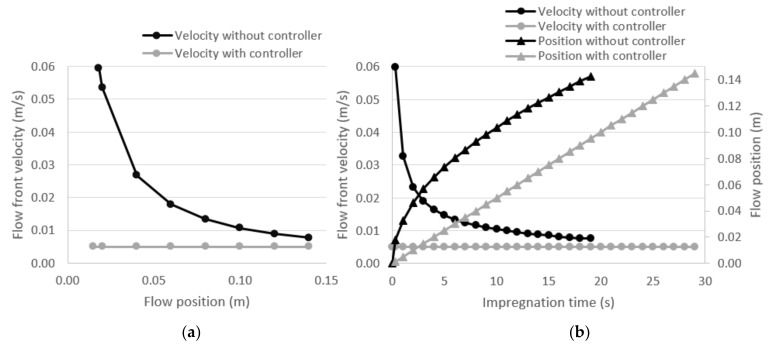
(**a**) Theoretical evolution of the flow front velocity as a function of flow position and the effect of adding the flow front controller; and (**b**) the theoretical evolution of the flow front velocity and position as a function of the process time and the effect of adding the flow front controller.

Equation (3) is plotted (Figure 1) as a numerical example to clarify the effects of adding the flow controller. Representative values of each parameter were used ($∆P_{valve}=1\;\mathrm{bar}$; μ=350 cP; $u_{opt}=5\;\mathrm{mm}/s$; ∅=0.4; $K=1.5\;\times \;10^{-9}$). As shown in Figure 1a, the flow front velocity is not linear with the flow position. Since the pressure gradient is changing as the flow distance increases, the flow front velocity decreases. According to Darcy’s model (Equation (2)), the velocity is proportional to the pressure gradient ∆P∆x. At the beginning of the process, the external pressure is applied over a very short distance of impregnated laminate. Then, as the flow goes further, the same external pressure is applied over longer impregnated distances as the pressure losses become higher. Consequently, the velocity is getting lower as the impregnated distance or process time goes further. This means that the void distribution will not be homogeneous through the laminate. Moreover, the mechanical properties will be non-homogeneous. The ideal flow front velocity is also presented in Figure 1a as a straight line showing a constant value. This last ideal situation will produce optimal and homogeneous mechanical properties. 

Furthermore, as described in Figure 1b, the flow front velocity is neither constant nor linear with the time. Since the void generation is highly dependent on the impregnation velocity, differences in void content will be found in the laminate. Additionally, most of the time, the impregnation velocity is far from the optimum value (i.e., with the controller), especially at the beginning and at the end of the process. Consequently, the mechanical properties will be negatively affected. In a similar way, the flow position is not linear with the time. It must be highlighted that the total impregnation time is higher in all cases when the controller is added. According to the Figure 1b, the position curve without a controller reaches the total length before the controlled case. Furthermore, the flow front velocity at the end of the process is higher than the optimum velocity. Otherwise, the last part of the laminate would occur at velocities lower than the optimum. In this context, due to a lack of linearities, the implementation of a flow controller is not as easy as the implementation of other techniques such as RTM, in which flow rate can be imposed as constant.

**Figure 2:**
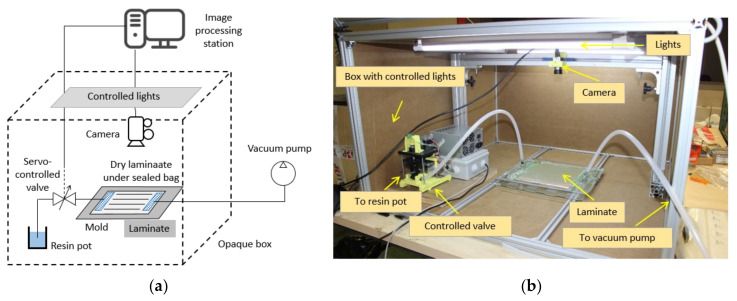
(**a**) Schematic of the setup; and (**b**) laboratory implementation.

To perform the experiments, a flow controller was added to a conventional setup. The whole setup is shown in Figure 2. A controlled valve was added in the resin tube between the resin pot and the laminate. An algorithm reads and processes the flow position data, then evaluates the instantaneous velocity and compares it with the optimum preset value. Thus, the valve position is continuously changing to match the optimum and the measured value.

In this implementation, a monochrome camera Mako U-130 (Allied Vision Technologies, Stadtroda, Germany) with 1.3 megapixels and a SSE0812NI lens were used (Securame SL, Barcelona, Spain). The controller was managed by a MATLAB GUI specifically developed for this application. It was based on a microcontroller and two servo-motors (Nema 17, GEMS, St. Paul, MN, USA) and Pololu A4988 drivers (POLOLU, Las Vegas, NV, USA). A controlled lighting system was also used to prevent any external influence during image recognition. Four 600 mm LED tubes of 10W (PHILIPS, Amsterdam, Netherlands) were used. The assembly was surrounded with a box of 900 × 900 × 500 mm^3^.

### 2.2. Specimen Manufacturing

For all the manufactured specimens, the process parameters remained unchanged with the exception of the flow front velocity which was set according to the target in each case. The applied vacuum pressure was in all cases −95.0 ± 0.1 kPa absolute pressure, measured in the vacuum line entering the mold. Curing was carried out at room temperature according to the resin manufacturer’s specifications. The resin was degassed prior to the infusion process to reduce the dissolved bubbles, according to Oosterom et al. [32]. For this purpose, the same vacuum pressure was applied for more than 2 min after mixing with the catalyst. Two stacks of 6 plies were tested: [0_2_, 90]_S_ and [90_2_, 0]_S_. Two additional layers of random fiber mat were placed in the middle of the laminate. These random plies were included to increase the impact properties and reduce the degree of biaxiality of the laminate. These configurations provide a reasonable thickness and stiffness, aiming to have a certain degree of inertia and strength in the two perpendicular directions of the plane. These configurations allow the evaluation of how laminates with a different nature and orientation can induce the generation of interlaminar and intralaminar voids. The dimensions of the fabrics were 300 × 250 mm^2^. Then, the specimens of 150 × 100 mm^2^ were cut using a circular saw. Stitched specimens were manufactured in a similar way. In this case, the stitching of [0_2_, 90]_S_ + 2 random mat layer laminates was performed using a single-side stitching process employing the CNC sewing device Pfaff 3574 (Pfaff, Karlsruhe, Germany). Specimens were arranged in pairs and adapted to the available frame sizes in the sewing machine. The stitching pattern was a squared grid with stitch spacing of 3.33 mm and 10 mm of seam distance, corresponding to a stitch density of 5 stitches/cm^2^. This value is in the range of most of industrial applications [33]. 

Target impregnation velocities were distributed around the optimum point observed in previously performed tensile tests [7] based on the available data in the literature. These are summarized in Table 1, among which the unstitched (US), stitched (S), different velocities (V1–V4) and the optimum velocity (VO) have been described. The optimum velocity was concluded from the preliminary test performed on [0_2_, 90]_S_ laminates. Taking into account the fact that stitching leads to the existence of resin-rich regions and places where macrovoids can appear, the chosen velocities for stitching were slightly higher than those for unstitched laminates.

To manufacture the specimens, a peel ply and a high-porosity layer (Dianet, with 135 g/m^2^ and 1.19 mm of thickness) were used. A 75 µm nylon-based vacuum bag was also employed (model BF-32 Wrightlon^®^ from Airtech Inc., Oldham, UK). Plane channels were used in the vacuum and resin ports (Diadrain 50 mm × 4 mm) and Ø9 mm–Ø12 mm hoses with polyethylene T-connections were set in the resin/vacuum ports. A two-stage vacuum pump Edwards^®^ 80 (Edwards Vacuum, Burgess Hill, UK) was also used. The adopted mold was a 900 mm × 700 mm steel sheet. For all specimens, e-type fiber glass plies of 600 g/m^2^ were used. Polyester resin Palatal^®^ P 4 TV-28 (Aliancys, Shaffhausen, Switzerland) was catalyzed with 2% weight medium-reactivity catalyst Curox^®^ M312 (United Initiators, Pullach, Germany). The dynamic viscosity of this system was 335 mPa·s at room temperature. The room conditions were kept at 25 ± 1 °C and 60 ± 5% of relative humidity during the manufacturing and testing. After manufacturing, the specimens were cut using a circular saw (brand Dremel, model MotoSaw, Breda, Netherlands).

The prepared specimens for the impact test were arranged as outlined below. Four specimens for each pre-set velocity were tested:

- Four groups of four specimens [0_2_, 90]_S_ without stitching at constant impregnation velocity;
- Four groups of four specimens [90_2_, 0]_S_ with stitching at constant impregnation velocity;
- Four group of four specimens [90_2_, 0]_S_ without stitching and manufactured at constant and optimum impregnation velocity, obtained from the previous specimen analysis.

The first group enabled identifying the optimum velocity. The second and third groups made it possible to evaluate the mechanical influence of stitching. Additionally, the second group was employed to define the optimum velocity for stitched laminates.

### 2.3. Testing Procedure

Tests were performed according to the ASTM D7136/ASTM D7136M standard [34]. Initially, the impactor final velocity at the center of the plates was roughly determined from the potential energy, assuming that the energy losses along the sliding on the columns was negligible. Then, a fine adjustment in the initial height was performed according to mounting transductors. 

The required initial velocity to produce perceptible damage was estimated according to similar published works and verified by preliminary tests. As a reference, Sutherland and Soares [35] used 100 × 100 mm^2^ and 200 × 200 mm^2^ and 3.5 mm-thick specimens made of polyester with glass fiber and found that 15 J produced noticeable delaminations in both plates. According to these references, the breaking mechanisms are highly dependent on the impact energy. Consequently, two different energy levels were used in the tests to cover the main damage mechanisms in the optimization process. First, 13 J was applied to all specimens. Subsequently, 26 J, which represents cumulative damage, was applied to the same specimens. After the tests, results were post-processed using a specially programmed MATLAB routine. Data were acquired from load cells at 250 kHz and 20 kHz for the two impact systems used. A CFC-second-order Butterworth filter [36] and a cut-off frequency of 6 kHz were also applied to reduce the effects of vibration during impact. 

In terms of results, three values were pointed out in the contact force curve [31]. On the one hand, the critical force (*F_c_*) or damage threshold load (*DTL*) was evaluated as that at which fracture was initiated. Subsequently, the contact force continued growing to a peak value, (*F_m_*), which was always higher than the critical value.

**Figure 3:**
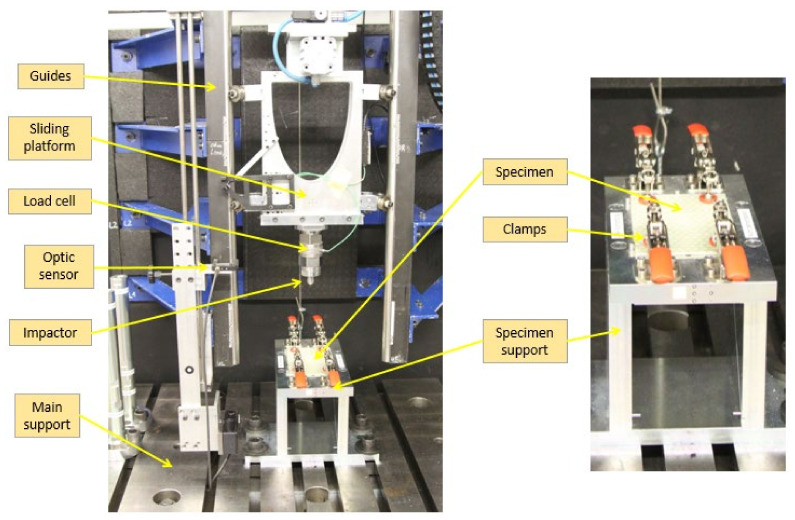
Setup for impact tests using the 15 m height impact test machine.

**Figure 4:**
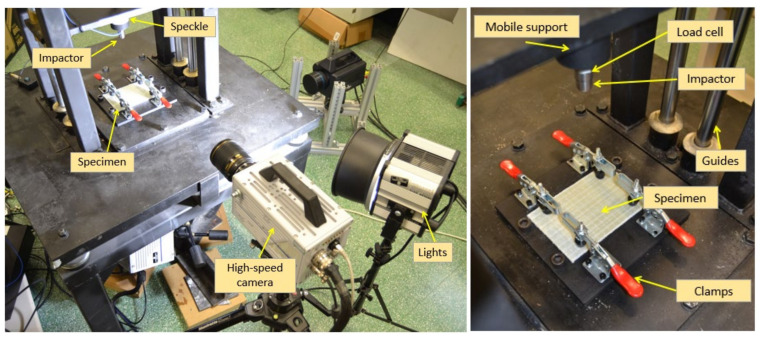
Setup for impact tests using the 2 m height impact test machine.

Two drop tower test machines were employed for impact testing: one 15 m high (Figure 3, Leibniz Institut for Composite Materials, Kaiserslautern, Germany), and a second one 2 m high (Figure 4, University of Jaén, Jaen, Spain). In both cases, the same impactor and energies were used. Specimens were positioned according to ASTM D7136/D7136M specifications and fixed to a solid steel base using four clamps. The impactor, a hemispherical Ø16 mm made in steel, was joined through the cell to the mobile structure with sliders on the rails. In the first machine, the suspended mass was 4312 ± 1 g, while 7750 ± 1 g was measured in the 2 m drop tower. The 15 m drop tower determined the impact velocity by a photometric barrier placed near the impact point. The signal processing directly reported the velocity value. For the 2 m drop tower, digital image correlation was employed, as other authors have previously [37]. A speckle pattern was applied to the moving components that moved with the impactor. A high-speed camera captured the falling motion of the assembly. The processing of the sequence allowed to calculate the instantaneous position of the rigid solid during the falling, and consequently, the velocity at the point of impact was evaluated. For this purpose, a high-speed camera FASTCAM S4A 1024 × 1024 CMOS (Photron, Tokyo, Japan) and an AF Nikkor 50 mm f/1.4D (Nikon Corporation, Tokyo, Japan) were used. The frame rate was set to 4000 fps and the shutter speed to 1/4000 s. This configuration showed sharpened images, as required to assess the impact velocity. The displacement field was evaluated using a commercially available software package (VIC-2DTM software from CORRELATED SOLUTIONS, INC. Irmo, SC, USA), and then, a MATLAB routine was used to calculate the velocity.

First, specimens were impacted with 13.0 ± 0.1 J in the 15 m drop tower (initial height of 0.329 m) and 2.46 ± 0.01 m/s as the impact velocity. Subsequently, the specimens were tested in the 2 m drop tower with an energy of 26.0 ± 0.2 J (initial height of 0.342 m) corresponding to velocities of 2.59 ± 0.01 m/s. After the impact, the moving structure was stopped in order to prevent successive impacts. The impactor force, position and velocity data, as well as the damaged area of each specimen, were subsequently analyzed.

In order to determine the damaged area after the impact events, an optical technique was employed. The effect of light transmission/diffusion through the specimens was used, taking into account the translucent nature of the polyester/fiberglass. A monochrome camera Mako U-130 (Allied Vision Technologies, Stadtroda, Germany), a light-emitting diode (LED) back-light source and the damaged specimen in the middle were set to take pictures of each specimen. An image-processing system based on filtering and segmentation techniques allowed an accurate damaged area measurement. Different authors have used this technique to estimate and compare the damaged area. Similar results were reported when compared with other complex and expensive techniques [26,38]. To calculate the damaged area, a MATLAB routine was specifically developed. For this purpose, segmentation techniques were previously calibrated in damaged and undamaged specimens. The application of classifiers [39] enabled the isolation of the damaged area from the rest of the details in the images. The objective was to take the reference intensity levels from each impacted specimen and compare them with the levels for the undamaged specimen, so that the difference corresponded to the damaged area. Firstly, the mean filter was applied to reduce the noise levels and the Sobel algorithm was used to detect the contour of the damaged area after binarization [40]. Then, segmentation was applied to measure the area and discard pointless information in the images. As a result, the damaged area was reported.

## 3. Results and Discussion

Each specimen was manufactured at a constant velocity using the velocity control system described in Figure 2. The velocity values given as input to the control system are described in Table 2. These values correspond to the mean values and the standard deviation found for each group of specimens. Small variations between the real velocity and the target were found. They are related to the calibration of the prototype system and other aspects such as the resolution of the optical system and the acquisition and processing rate. Since the deviations are relatively small, the discussion will refer to the set point values.

**Figure 5:**
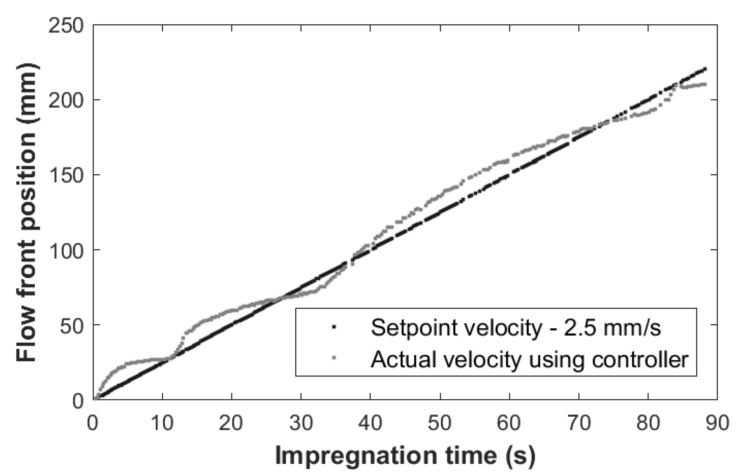
Impregnation process. Flow front position in [90_2_, 0]_S_ stitched specimen: the target of 2.5 mm/s (grey) and the real value (black).

As an example, Figure 5 shows the controlled impregnation of the stitched specimens at 2.5 mm/s. The curve in grey shows the actual flow position measured by the control system, which shows an almost linear change during the whole impregnation process. The target position is shown in black as a straight line which implies a velocity of 2.5 mm/s. The similarity between one curve and the other was numerically assessed by the Kolmogorov–Smirnov test. It reported a dissimilarity value of 0.02 < 0.05, allowing to state that both curves can be considered similar to each other. The rest of the laminates showed similar equivalences.

**Figure 6:**
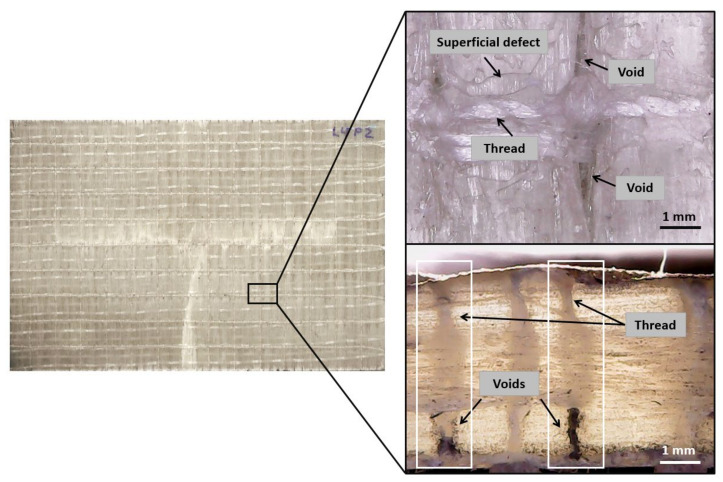
Impregnation defects in stitched laminates. The applied vacuum generates voids around the seams (**top**) and the cross-section of stitched laminate near the stitching point (**bottom**).

After the impregnation and curing process, the visual inspection of the surface of the stitched laminates showed a clear trend to generate voids around the stitches (Figure 6). The induced distortion in the yarns by stitching enhanced the creation of dry areas. These voids are approximately 1 mm wide and several millimeters long, and they progressively dissipate from the stitching point. It is well known in the literature that stitching generates resin-rich zones, although this is not related to the appearance of large superficial voids that can spread through the entire thickness of the laminate. In this specific case, it should be highlighted that stitching is usually covered with additional elements such as mat ply to homogenize the surface. In these cases, the voids shown in Figure 6 may be filled with resin, which may increase the weight and the possibility of the generation of hidden voids. In other cases, where RTM is used as the impregnation process, the positive pressure generated in the cavity encourages these cavities to be filled with resin. However, in the infusion process, the vacuum application can enhance the generation of areas that contain a large number of voids, as shown in Figure 6.

**Figure 7:**
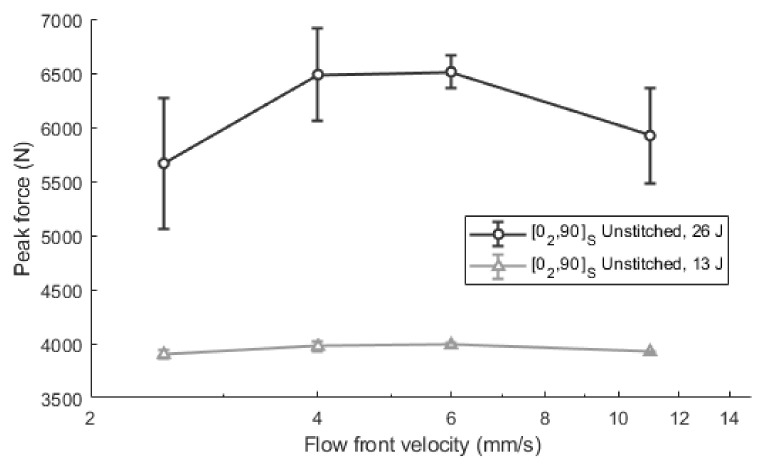
Maximum impact force in unstitched [0_2_, 90]_S_ laminates at 13 J and 26 J.

Regarding the mechanical results, the maximum impact forces for the [0_2_, 90]_S_ laminates at 13 J and 26 J are summarized in Figure 7. In the 13 J impacts, there is a slight reduction in the peak force values as the velocity value is getting away from the optimum value. In the range of analyzed values, there is an increase in the maximum reaction force from 3899.4 ± 38.2 N, when the impregnation speed is very low (2.5 mm/s), to 3999.1 ± 15.6 N at 6.0 mm/s, which means an increase of approximately 2.6%. In a similar way, when the velocity changes from 11.0 mm/s to 6.0 mm/s, the maximum force increases by approximately 1.9%, from 3924.4 ± 9.8 N to 3999.1 ± 15.6 N. In impacts at 26 J, more remarkable variations were observed when comparing the results at optimum values and those at very low or very high velocities. Thus, the peak force improves from 5665.4 ± 608.3 N at 2.5 mm/s to 6512.0 ± 150.8 N at 6.0 mm/s (an increase of 14.9%) and from 5922.6 ± 444.8 N at 11.0 mm/s to 6512.0 ± 150.8 N at 6.0 mm/s (an increase of 10.0%). Hence, the optimum range for impacts at 13 J is slightly defined between 4 mm/s and 6 mm/s. The same values can be deduced from the impacts at 26 J, although in this case, a clearer definition is shown. Minor improvements were reported for low impact energies, because the effect of voids during the crack propagation is also reduced. In these cases, the energy is not sufficient to overtake the crack initiation threshold.

**Figure 8:**
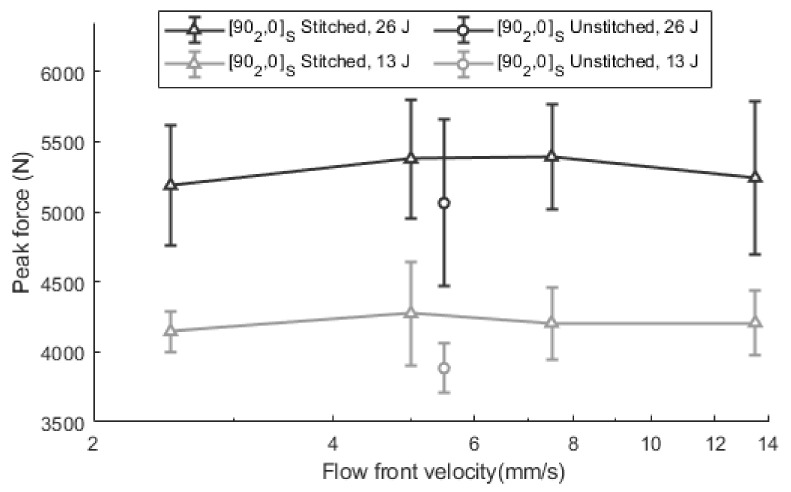
Maximum impact force in [90_2_, 0]_S_ in stitched and unstitched laminates at 13 J and 26 J.

Moreover, the maximum impact forces for the [90_2_, 0]_S_ laminates at 13 J and 26 J are summarized in Figure 8, which includes the unstitched laminate at optimum velocity as a reference. Statistically significant improvements were observed when stitching is added at both energy levels. Maximum values are increased from 3884.2 ± 177.5 N to 4274.3 ± 370.6 N in impacts at 13 J, and from 5061.1 ± 595.6 N to 5390.9 ± 373.4 N in impacts at 26 J, which means increases of 10.0% and 6.5%, respectively. The highest forces were observed at intermediate velocities, 5.0 mm/s and 7.5 mm/s. Specifically, at 13 J, the maximum is shown at 5.0 mm/s and for the case of 26 J, the maximum is between 5.0 mm/s and 7.5 mm/s. This means an increase of 3.1% when the velocity is optimized for 13 J impacts and around 3.9% for 26 J impacts, when the values are compared to non-optimized velocities. A wide dispersion in the values is observed in all cases, although it is slightly smaller in the 13 J scenario. Such dispersion, especially at 26 J, can be associated with the fact that the impact is performed on previously impacted specimens at 13 J. Specimens accumulate random effects in the second test because of small differences in the specimen’s positioning as well as the recovery of elastic deformation from the first impact or creep effect, which reduce the repeatability of the results.

When the unstitched and stitched specimens are compared, the stitched laminates do not show such a marked change between the peak forces at extreme velocities and those that could be considered optimum. This effect could be associated with the fact that the influence of stitching is so high that it attenuates the effect of voids—including both micro and macro voids.

**Figure 9:**
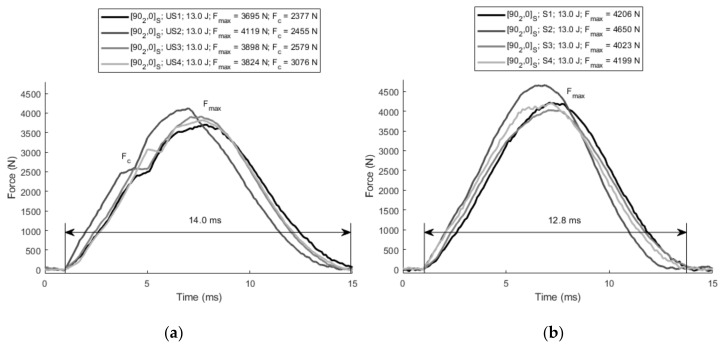
(**a**) Force in unstitched [90_2_, 0]_S_ laminates at 13 J; and (**b**) force in stitched [90_2_, 0]_S_ laminates at 13 J.

As shown in Figure 8, the unstitched specimens show a remarkable reduction in the impact properties when they are compared with the stitched specimens. In relative terms, the peak force of the unstitched specimens is approximately 90–95% of those which are stitched. The reason behind this reduction is the existence of induced damage even at low energy levels which generates a stiffness reduction. As shown in Figure 9a, all unstitched specimens show critical forces (*F_c_*) between 2500 N and 3000 N, which represents approximately 60% of peak forces. This means that noticeable damage was generated in these cases. In contrast, all stitched specimens impacted at 13 J showed a sub-critical impact, which shows that the stitching has a positive effect, as it has protected the laminates from appreciable damage. In contrast, when stitching is added to the previous laminates (Figure 9b), the curves do not show any critical point.

From the point of view of stiffness, significant differences were also observed between the unstitched and stitched laminates. The average impact duration was reduced from 14.0 ± 0.6 ms to 12.8 ± 0.5 ms when stitching was added. It should be highlighted that the stitched specimen S2 shows slightly higher force as well as shorter impact duration (11.5 ms) than other stitched specimens, which means higher dynamic stiffness. This specimen was manufactured at 5 mm/s, which corroborates the fact that the optimum impregnation velocity is close to this value.

When specimens are tested at 26.0 J, the behavior becomes supercritical in all cases. The duration of the impacts is also shorter for stitched laminates and thus, a higher stiffness is deduced. The mean impact duration of the specimens with stitching was 15.1 ± 1.9 ms, while for specimens without stitching, this was 16.4 ± 2.5 ms. Similar trends in stiffness and peak force were also reported by [41] when comparing stitched and unstitched laminates. Regarding the maximum impact force values, no significant differences were observed. On average, 5275.3 ± 647.8 N was obtained for unstitched laminates, while 5340.3 ± 727.6 N was concluded for stitched laminates. No significant differences (0.06 > 0.05) were concluded for the critical forces, and 4795.3 ± 1253.5 N and 4964.63 ± 451.4 N were obtained for the unstitched and stitched laminates, respectively. 

**Figure 10:**
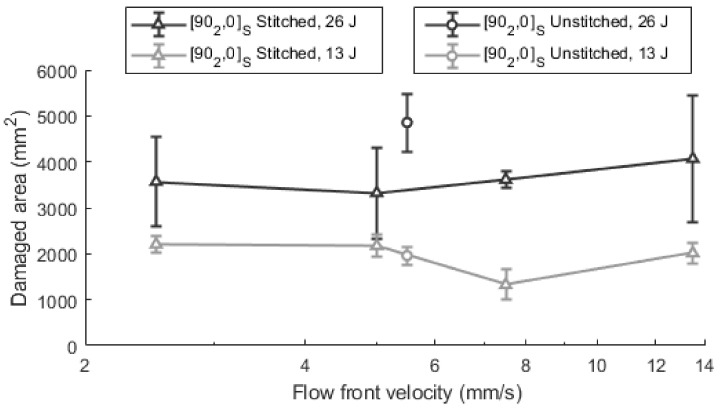
Damaged area for stitched and unstitched [90_2_, 0]_S_ laminates at 13 J and 26 J.

Similar conclusions can be deduced from the damaged area analysis. Figure 10 shows the results of damaged area for unstitched and stitched [90_2_, 0]_S_ laminates. When the impregnation velocities are analyzed for the stitched laminates, optimum values can be deduced. At 26 J, the minimum is shown at 5.0 mm/s, and the damaged area was 3324 ± 992 mm^2^. The relative reduction from 11.0 mm/s (4069 mm^2^) to 5.0 mm/s was approximately 18.3%. For the impacts at 13 J, the minimum damaged area was found at 7.5 mm/s (1322 ± 329 mm^2^). The relative reduction from extreme velocities to the optimum velocity is approximately 40.0%. These results agree with the conclusions of other authors such as Ricotta et al. [42], who found that fracture toughness decreases as the void content increases. Consequently, minimum damage is observed when the void content is minimized.

Noticeable differences are shown between the stitched and unstitched laminates for 26 J impacts, but this effect was not observed at 13 J. At 26 J, the damaged area was 4858 ± 628 mm^2^ in the unstitched laminates while 3324 ± 992 mm^2^ was measured at the optimum velocity for stitched laminates which represents a reduction of 31.6% when stitching is added. For 13 J impacts, the effect of stitching is negligible, as the value of 1958 ± 196 mm^2^ is in the curve defined in the range from 5 mm/s to 7.5 mm/s. Then, for low energy impacts, both types of laminates behave in a similar way and stitching has no relevance. Indeed, as pointed out by [33], there are no remarkable differences in damage at low impact energies. This is because in the low energy impacts in the analyzed materials, the damage is not extended by more than 10–20 mm. In the particular case of the used material, the distance between seams is 10 mm, which is within the range of crack lengths generated at 13 J. In such cases, delaminations have approximately the same size in both stitched and unstitched materials. At the delamination scale, both materials are closely similar. In this sense, stitching does not prevent small cracks from occurring and propagating. Then, it can be concluded that the effect of adding stitching at low-energy impacts is not significant. When the applied energies are higher, delaminations tend to propagate beyond the seam distances. In these cases, stitching can prevent delamination from propagation, as it works as a link between adjacent layers. 

**Figure 11:**
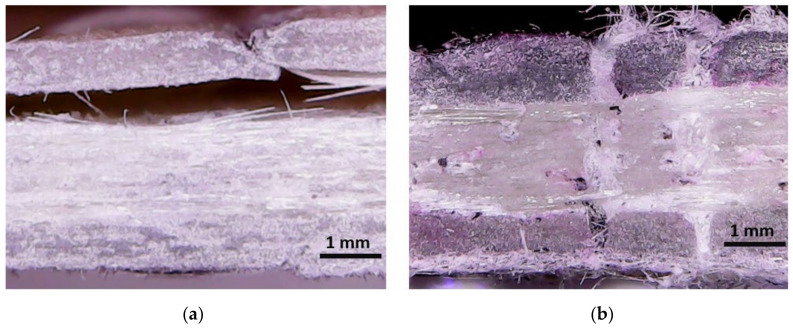
Damage after 26 J impact in: (**a**) an unstitched laminate where the delaminations reach the specimen edges; and (**b**) cross-sectional view of stitched laminate where delamination was avoided.

After a thorough inspection of the tested specimens, delaminations show remarkable differences when stitching is added, as shown in Figure 11. In the unstitched laminates, the delamination is extended over the edge of the specimen (Figure 11a). The lack of links between adjacent layers promotes large interlaminar delaminations. Compression loads at the impact side lead to buckling and debonding effects at the top layers. Different orientations of the plies intensify these delaminations as high shear stress is generated due to the mismatch stiffness at the interface [43]. In contrast, a cross-sectional view of a stitched laminate is shown in Figure 11b. Although the amount of void in this specimen is higher, especially at the stitch area, the delamination is prevented by the transverse stitching threads which are working axially. In these cases, the damage is propagated through adjacent stitches. Initially, the tensile stress at the bottom promotes delamination [44] although the stitches restrain its propagation. These differences have a remarkable effect in the impact behavior. As highlighted above, stitched laminates reportedly increase impact forces by up to 10.0%. Despite of the amount of void, the reduction in the delaminations, as shown in Figure 11b, reported the minimum damaged area. Similar results were reported by Tan et al. [27] for stitched and unstitched laminates after impact loads.

**Figure 12:**
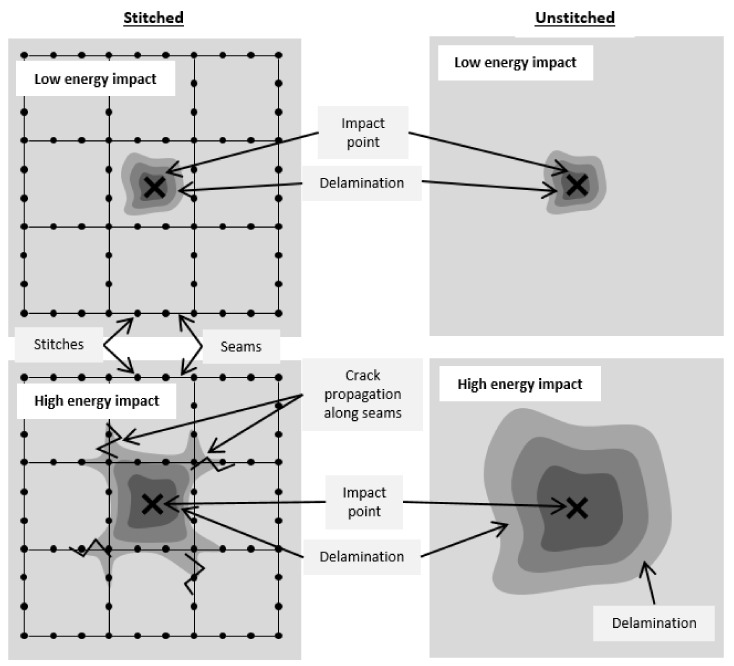
Schematic comparison between the generated damage in the stitched (**left**) and unstitched laminates (**right**), impacted at low (**top**) and high energy (**bottom**).

The scheme shown in Figure 12 summarizes the results of the stitched and unstitched specimens. With impacts of 13 J, the induced delamination is smaller than the characteristic seam distance. In these cases, stitching may not have a relevant effect and the damaged area is closely similar in both configurations (Figure 12, top). For impacts of 26 J (high energy impacts), the damage is extended beyond the seam distances and delaminations are radially propagated around the impact point (Figure 12, bottom). This is the result of high interlaminar delaminations. 

It can be concluded that stitching does not prevent delaminations occurring but it limits their propagation beyond the characteristic dimension of the stitching pattern (Figure 12 bottom, left). Similar conclusions were reported by Tan et al. [27]. At high energies, stitches could exceed the load limit and the delamination will be propagated to the next seam. Consequently, the interlaminar stress will be redistributed and the delamination will be limited. Then, stitching is especially interesting for high impact energies and the flow front optimization could provide improved impact behavior.

## 4. Conclusions

A novel manufacturing methodology was applied to optimize the impact properties of stitched and unstitched laminates in the resin film infusion (RFI) process. It was based on the control of the impregnation velocity which can reduce the void content.

The main findings are summarized below:

- Impact properties were improved in both stitched and unstitched laminates by optimizing the flow front velocity. The peak force during the impact event and the damaged area were analyzed at two energy levels;
- When the peak forces are analyzed, the same optimum range of impregnation velocities is concluded for different fiber orientations. No significant differences were found in the optimum velocity for unstitched and stitched laminates. For high energies, significant increases in the peak force up to 14.9% were observed. Minor improvements were reported for low impact energies, because the effect of voids during the crack propagation was also reduced. In all cases, the range for optimum velocities was set between 5 mm/s and 7.5 mm/s;
- Damaged area analysis supports the peak force analysis conclusions, and the same optimum range was deduced and statistically significant. The relative reduction in the damaged area can reach 40.0%. Then, the “process window” for these materials can be set between 5 mm/s and 7.5 mm/s;
- At optimum values, stitched laminates reported the highest impact properties as well as the minimum damaged area. While unstitched specimens showed critical impacts, subcritical impacts were observed in stitched cases;
- During the impregnation of stitched laminates, several voids and empty areas were observed around the stitch point. These may be associated with the use of high vacuum pressures and further analysis is required;
- Stitching does not affect the delaminations for low impact energies. For high impact energies, the average delaminated area can be reduced by 31.6% if stitching is added. In this sense, stitching is highly effective for high energy levels. In these cases, stitch points prevent the propagation of delamination.

By this proposed methodology, the resin infusion process will reduce the influence of the operator’s skills, and the impact properties and dispersion in the results will consequently be improved. Thus, a reduction in weight is achieved by two ways: improved mechanical properties mean a reduction in the required amount of material and weight; and a reduction in the dispersion of the results allows the use of reduced safety coefficients and therefore less material.

## Figures and Tables

**Table 1:** Flow velocities used as a target for manufacturing.

Unstitched Laminates [0_2_, 90]_S_		Stitched Laminates [90_2_, 0]_S_	
Group—Velocity	Target Velocity (mm/s)	Group—Velocity	Target Velocity (mm/s)
US—V1	2.5	S—V1	2.5
US—V2	4.0	S—V2	5.0
US—V3	6.0	S—V3	7.5
US—V4	11.0	S—V4	13.5
		US—VO	5.5

**Table 2:** Target and real impregnation velocities for the manufactured specimens.

Ref.	Target Velocity (mm/s)	Real Velocity (mm/s)
US—V1	2.5	2.5 ± 0.7
US—V2	4.0	4.1 ± 0.5
US—V3	6.0	5.9 ± 1.8
US—V4	11.0	11.3 ± 0.5
S—V1	2.5	2.5 ± 0.7
S—V2	5.0	5.2 ± 0.7
S—V3	7.5	7.3 ± 1.6
S—V4	13.5	13.7 ± 0.7
US—VO	5.5	5.6 ± 0.6
